# Online Misleading Information About Women’s Reproductive Health: A Narrative Review

**DOI:** 10.1007/s11606-024-09118-6

**Published:** 2024-11-07

**Authors:** Jennifer N John, Sara Gorman, David Scales, Jack Gorman

**Affiliations:** 1 Penn Medical Communication Research Institute, University of Pennsylvania, Smilow Center for Translational Research Room, 12-136, 3400 Civic Center Boulevard, Philadelphia, PA 19104-5160 USA; 2 Critica, Bronx, NY USA; 3 Weill Cornell Medicine, New York, NY USA

**Keywords:** reproductive health, women’s health, misinformation, patient education, social media

## Abstract

Misinformation about reproductive health threatens to harm health outcomes, compromise medical trust, and enable misinformed policy restrictions. In recent years, reproductive health misinformation has proliferated online due to ideological campaigns and limited content moderation for reproductive health topics. Developing evidence-based practices to counter reproductive health misinformation requires an understanding of the content that women are exposed to online, which is currently lacking. This review sought to identify common claims and narratives about reproductive health on social media and the internet that could easily mislead. We performed a narrative review of articles about online reproductive health misinformation, from which we extracted misleading claims and narratives. We conducted a qualitative content analysis to describe the ways in which the claims and narratives could be misleading. We found that potentially misleading claims and narratives about reproductive topics relating to contraception and abortion, fertility, chronic disease, breast cancer, maternal health, and vaccines abound across social media platforms and websites, with 112 identified in total. One-third of this content could mislead by claiming that evidence-based interventions were associated with unattributed risks. Twenty-three percent made medical recommendations that do not align with professional guidelines. Fourteen percent promoted alternative medicine. Smaller numbers of claims and narratives exaggerated risks of medical interventions, discouraged evidence-based interventions, directly undermined medical trust, and proposed inaccurate biological mechanisms. Healthcare professionals can proactively promote evidence-based medical decision-making by increasing their awareness of prominent misleading claims and narratives.

**Supplementary Information:**

The online version contains supplementary material available at 10.1007/s11606-024-09118-6.

## INTRODUCTION

Health information that is misleading or false, also known as misinformation,^1^ is increasingly recognized as a threat to optimal health outcomes.^2^ Many patients now seek health information online, which may contradict recommendations from their healthcare providers. Although reproductive health is a frequent target of misinformation, it has received substantially less attention compared to topics such as COVID-19 and vaccines.^3^ For example, more than half of endometriosis patients in a survey believed that someone with endometriosis could never become pregnant after seeing the false claim online.^4^ In a survey, 30% of healthcare providers held misconceptions about the safety of intrauterine devices (IUDs) for nulliparous women,^5^ even though the American College of Obstetricians and Gynecologists^6^ considers IUDs a first-line contraceptive method for adolescents.

The stakes of reproductive health misinformation are high. False claims have been incorporated into policy that restricts reproductive rights and threatens patient safety. For example, the Supreme Court case *Gonzales v. Carhart* reinforced a ban on “partial-birth abortion,” a medically inaccurate term intended to perpetuate stigmas and misconceptions.^7^ In this case, Justice Anthony Kennedy claimed without evidence that women can develop severe depression after an abortion.^8^ Ohio House Bill 413 proposed that physicians should reimplant ectopic pregnancies in the uterus, a medically impossible procedure that could delay life-saving treatment.^9^ Misinformation can influence consequential health decisions: female community college students who believed contraception causes infertility were less likely to use hormonal contraception.^10^

A variety of factors have led to an information environment in which misinformation about reproductive health proliferates. Political and ideological campaigns exploit misinformation;^11^ for example, crisis pregnancy centers give women false information about abortion.^12,13^ Reproductive health is understudied, leading to information vacuums that are conducive to misinformation.^14^ Gendered sociocultural constructs could increase the acceptability of false claims that align with these narratives,^15^ and misleading narratives often exploit medical distrust among women who do not feel supported by the healthcare system.^16^ While the internet has allowed for open discussions of reproductive health, online platforms have enabled an unprecedented amplification of misinformation.^2^ Unlike other public health topics such as COVID-19, social media moderation of unreliable reproductive health content is minimal.^17^ Inconsistently enforced content moderation policies have resulted in evidence-based information about abortion being removed from online platforms.^18^

We sought to review the literature on online reproductive health misinformation and to systematically categorize the types of misleading claims and narratives that have been reported to prepare clinicians to respond to misinformation.

## METHODS

We pursued a narrative review of research articles and reports on the topic of online reproductive health misinformation.^19^ We selected a narrative review approach given our interest in misleading narratives (in addition to strict misinformation), which are not easily identified through keyword searches due to the importance of context and implied meanings. Furthermore, research methodologies in this area are highly varied, and a range of terms are used to describe reproductive health misinformation. A systematic review would thus be too restrictive in its focus. We followed the hermeneutic approach to literature reviews, which “emphasizes continuous engagement with and gradual development of a body of literature during which increased understanding and insights are developed.”^20^

We primarily included peer-reviewed articles identified mainly through PubMed and Google Scholar using search terms relating to women’s reproductive health topics (e.g., “endometriosis,” “ovarian cancer”) based on the Office on Women’s Health index of health topics (Table 1).^21^ We included topics that pertain to women’s health broadly (e.g., breast cancer) as well as reproductive health, as we anticipated salient thematic overlap across these topics. To be consistent with other literature and given gendered dynamics relating to reproductive health, we focus on medical issues affecting individuals assigned female sex at birth. We acknowledge that transgender women have unique reproductive health experiences, which are outside of the scope of this review.

**Table 1:** Reproductive Health Search Terms

Contraception
Abortion
HPV vaccine
Covid vaccine fertility
Sexually transmitted infections
Pregnancy
Childbirth
Maternal health
Postpartum
Fertility
Infertility
Breast cancer
Gynecological cancer
Reproductive cancer
Cervical cancer
Uterine cancer
Ovarian cancer
Vaginal cancer
Vulvar cancer
Sexual function
Endometriosis
Polycystic ovarian syndrome
Menopause
Women’s health
Reproductive health
Bacterial vaginosis
Bladder pain syndrome
Mastectomy
Mammography
Chlamydia
Douching
Fibroids
Folic acid
Female genital mutilation
Female genital cutting
Herpes
Genital warts
Gonorrhea
Hysterectomy
Menstruation
Ovarian cysts
Pap
Pelvic inflammatory disease
Pelvic organ prolapse
Prenatal
Premenstrual syndrome
Syphilis
Trichomoniasis
Incontinence
Urinary tract infection
Yeast infection

These reproductive health topics were cross-referenced with terms referring to the internet and/or misinformation (e.g., “social media,” “myths”). Where relevant, we included references embedded in these articles. We also incorporated several gray literature reports and research blog posts from leading internet research organizations and articles known to the authors to be relevant that provided insights on the characteristics and spread of online misleading reproductive health information. Articles mainly consisted of content analyses of social media platforms and websites. Articles were reviewed to the point of thematic saturation, at which point the researchers determined that the major themes had been surfaced and additional articles were unlikely to contribute further. Forty-seven articles were included in the final analysis.

Articles were included if they were published between 2010 and 2024, were English-language and focused on English content on social media and other internet platforms, and discussed specific misleading claims and narratives about women’s reproductive health. Articles were excluded if they were published outside of the date range, were not in English or studied non-English content, did not address women’s reproductive health (e.g., articles about male contraception), and did not describe a specific misleading claim or narrative (e.g., articles that reported on the overall prevalence of misinformation or the accessibility of the content).

One author (JNJ) extracted specific misleading claims and narratives from each article by reviewing the relevant tables, figures, and text. To define misleading claims and narratives, we adapted the following definition of scientific misinformation to health topics: “publicly available information that is misleading or deceptive relative to the best available scientific evidence or expertise at the time and that counters statements by actors or institutions who adhere to scientific principles”;^1^ we note that varying terminology is used to describe health misinformation. This definition allowed us to include narratives with the potential to be misleading or undermine medical expertise, even if no falsifiable claims were made (e.g., exaggerating side effects from contraception). We included narratives because they can lead to healthcare-seeking behaviors that harm health outcomes, such as by undermining medical trust. Narratives typically transcend any particular claim, making it necessary to consider them separately. Studying narratives illuminates overarching themes relevant to many specific claims, enabling more broadly effective responses addressing a wider range of misinformation. We determined whether claims and narratives were misleading by reviewing the relevant medical literature, consulting professional society guidelines, and speaking with consultants with relevant expertise.

To characterize the claims and narratives, we identified broad categories according to medical topics and nested sub-topics. We additionally developed classifications to describe the way in which a claim or narrative was misleading. To do so, two authors (JNJ and JMG) separately inductively coded the entire dataset. We then compared and resolved discrepancies through a discursive, consensus-driven process, which yielded the final coding chart. One author (JNJ) conducted a second pass of coding using the jointly developed coding chart, and JMG reviewed this coding to confirm its accuracy.

## RESULTS

Forty-seven articles were included in the final analysis. Articles most often examined websites (36%), followed by TikTok (15%), Twitter (13%), YouTube (11%), and Facebook (9%). Smaller numbers of articles studied generative artificial intelligence chatbots (ChatGPT and Google Bard AI), Instagram, Quora, Pinterest, and various blogs and online forums. These articles yielded 112 unique misleading claims and narratives (Appendix Table 1). We identified six reproductive health topics describing the focus of the claims (contraception and abortion, vaccination, maternal health, fertility, breast cancer, and chronic disease), and an “other” category. The claims and narratives were further categorized based on specific sub-topics (e.g., contraception, endometriosis).

Eight codes describing types of misleading statements were developed (Table 2). The most commonly applied code was Unattributed Risk (32%), which refers to purported side effects or harms of a medical intervention that are not supported by medical evidence. Not Aligned with Professional Guidelines describes recommendations about the administration or use of medical interventions that go against or are not supported by current medical practice guidelines and was the next most frequent code (23%). Promotes Alternative Medicine content (14%) encourages the use of therapeutics or interventions that fall outside of mainstream medicine and for which there is no evidence, such as promoting the herb ashwagandha (an abortifacient) to improve prenatal mental health rather than antidepressants. Exaggerated Risk content (8%) presents a risk known to be associated with an evidence-based intervention as more common than it is. Eight percent of content was coded as Discourages Evidence-Based Interventions, which included recommending the refusal of such interventions or conveying negative attitudes toward them. Five percent of statements contained content that explicitly undermined medical trust by indicating that medical experts or institutions provide medical information or interventions that are suspect. Other claims and narratives also contained themes of mistrust by casting doubts about advice, guidance, or recommendations given by medical experts, but they were categorized according to the most directly stated claim. Inaccurate Biological Mechanism, such as claims that IUDs primarily prevent implantation, was found in 4% of claims and narratives. Five percent of statements did not fall into any of these categories and were classified as Other. All statements were assigned one code only.

**Table 2:** Codebook to Categorize Types of Misleading Claims and Narratives

Code	Definition	Frequency	Example
Unattributed risk	An evidence-based intervention is claimed to be associated with a risk for which there is no evidence.	32.1%	Plan B and Ella cause ectopic pregnancy.
Exaggerated risk	A risk known to be associated with an evidence-based intervention is presented as more common than it is.	8.0%	Hormonal contraception harms mental health.
Inaccurate biological mechanism	False claims about a biological phenomenon or mechanism of action of a therapeutic.	3.6%	Endometriosis can be caused by excess estrogen or ferritin.
Not aligned with professional guidelines	Recommendations about the administration or use of medical interventions that go against or are not supported by current medical practice guidelines. Includes inaccurate contraindications. Distinct from “promoting alternative medicine” in that there is not a component of a non-mainstream medicine intervention.	23.2%	Women of all ages, not only those actively trying to conceive, should check their AMH levels.
Discourages evidence-based interventions	Recommends against or conveys negative attitudes toward evidence-based interventions. Includes claims that underestimate efficacy of interventions.	8.0%	Women should decline oxytocin administration.
Promotes alternative medicine	Recommendations to use therapeutics or interventions that fall outside of mainstream medicine and for which there is no evidence.	14.3%	Patients with primary ovarian insufficiency should consume foods with antioxidant and anti-inflammatory properties.
Other	Any content that does falls outside of the above categories.	5.4%	Androgenic hair growth and sensory issues can be signs of endometriosis.
Undermines medical trust	Narratives and claims that explicitly fuel mistrust in medical and public health professionals, institutions, and systems.	5.4%	Doctors can’t be trusted to give reliable information about psychotropic medications during pregnancy.

Articles varied in whether and how they reported the frequency of a given claim or narrative. Many provided the percentage of posts that contained the claim or narrative, while others reported the total number of posts that included it. Some did not provide any measurements of the amount of misinformation.

### Contraception and Abortion

Misleading statements about contraception and abortion predominantly presented inaccurate or exaggerated risks. Online content frequently claimed that contraception causes weight gain, mental health issues, and infertility. Adverse side effects, both actual and unattributed, were often emphasized to a substantially greater extent than benefits.^22–30^ In one study, these harms were conveyed through narratives about contraception being “unnatural.”^22^ Misleading claims about contraception either referred to hormonal methods generally or focused on specific methods, most frequently LARCs including IUDs.^31^ Cycle-tracking was promoted inaccurately as an effective “natural” alternative on YouTube, failing to include critical caveats about its efficacy.^22^ Multiple studies found untrue online claims that abortion is associated with preterm birth, breast cancer, mental health issues, and infertility.^12,32–34^ This misinformation was frequently presented on the websites of crisis pregnancy centers. Promotion of abortion pill reversal, a procedure not supported by medical evidence, was found in large volumes across social media platforms and websites.^17,32,34–36^ Content encouraged women to use herbs and other “natural” methods to induce an abortion.^35,37,38^ One study found that ChatGPT exaggerated the risks of self-managed medication abortion, while providing accurate information about clinician-managed medication abortion.^39^ Generative artificial intelligence chatbots also made a range of false claims about managing a medication abortion.^40^

### Vaccination

A large number of studies examined online misinformation about vaccines against the human papillomavirus (HPV). This misinformation most often presented unsubstantiated risks allegedly associated with the vaccines, including increased sexual activity and death.^41–44^ Online content about COVID-19 vaccines focused on the unfounded risks of infertility and miscarriage.^38,45^ Claims about vaccination during pregnancy also emphasized the unsupported risk of miscarriage. Narratives undermined trust in recommendations regarding maternal vaccines made by the US Food and Drug Administration (FDA), the Centers for Disease Control and Prevention (CDC), and other health agencies.^46,47^

### Maternal Health

Misleading claims about maternal health related to pregnancy, miscarriage, childbirth, and breastfeeding.^48,49^ Overall, this content recommended alternative interventions that often conflicted with medical guidelines. For example, social media videos discouraged the use of injectable oxytocin (Pitocin) and epidural analgesia during labor and recommended herbal supplements or more than the recommended amount of weight gain or loss during pregnancy.^50–54^ Some narratives accused drug companies and medical professionals of providing unreliable information about psychotropic medications during pregnancy and encouraged pregnant women to reject their guidance.^54^

### Fertility

Fertility-related misleading claims primarily exaggerated the efficacy of interventions, evidence-based and not, to assess and improve fertility. For example, of the in vitro fertilization (IVF) outcomes depicted on TikTok, 89.3% were live births, a far greater proportion than in the general population.^55^ Specific claims about IVF made in these videos, however, were generally accurate.^55^ Websites addressing oocyte cryopreservation omitted information about limitations to the procedure, including that multiple cycles of egg retrieval may be needed and that frozen eggs may not result in a viable pregnancy.^56^ One study noted that websites selling direct-to-consumer anti-Mullerian hormone (AMH) testing made unsupported claims about the ability of AMH levels to predict fertility and menopause.^57^

### Breast Cancer

While none of the included studies addressed reproductive cancers, breast cancer was a common topic. On X (formerly Twitter), posts were found to describe unsupported risks of mammography separate from legitimate scientific debates about screening practices and to promote “natural” approaches to breast cancer screening aligned with the alternative health movement.^58^ Online content encouraged alternative breast cancer treatments, such as diet and supplements including colloidal silver and slippery elm, either as add-ons to traditional treatment or replacements.^59,60^

### Chronic Disease

Studies examined online claims about endometriosis, polycystic ovarian syndrome (PCOS), and premature ovarian insufficiency (POI). Misinformation about prevention, treatment, or cures with natural interventions such as diet and herbs were present for all three conditions.^4,61–63^ Misleading claims and narratives about endometriosis additionally undermined trust in healthcare and claimed that pregnancy was impossible with endometriosis.^4,62,64^

### Other

One study described YouTube videos about pelvic organ prolapse that emphasized surgical management over conservative treatment options.^65^

## DISCUSSION

This review found that misinformation about a wide range of reproductive health topics abounds online. At a time when access to reproductive healthcare and accurate information is impeded by significant legal and sociocultural barriers, misinformation from online sources could instead shape health beliefs. This possibility is concerning given that many of the misleading claims analyzed in this study directly or indirectly discourage the use of evidence-based medical interventions. For example, young women who turn to TikTok to learn about reproductive health may come away with the impression that hormonal contraception unequivocally compromises their health goals and that natural family planning is an equally reliable option. The subsequent contraceptive behaviors may result in adverse maternal and child health outcomes, particularly in states where abortion access is restricted.

The findings of this review can empower clinicians to protect patients from reproductive health misinformation. Just as clinicians take into account patients’ physical environments as social determinants of health, it is important to consider information environments.^66^ Being aware of the claims patients may encounter online is a critical first step for clinicians to engage in productive conversations about misinformation. For example, clinicians providing contraceptive counseling may wish to pre-emptively address concerns about weight gain or mental health. Similarly, if a patient inquires about AMH testing to predict fertility, their clinician may benefit from the knowledge that they may have been influenced by websites advertising direct-to-consumer testing. The prevalence of misinformation suggests that clinicians should consider assessing patients’ current beliefs and knowledge about a health topic before providing standardized patient education.

Patients who do not have access to a trusted clinician or who are accessing care outside of a clinical setting can build their resilience to misinformation by consulting reputable sources such as the American College of Obstetricians and Gynecologists, the Office on Women’s Health, and the Centers for Disease Control and Prevention. Digital health literacy practices to evaluate the credibility of online messages and messengers can protect patients against false claims.^67^ Other efforts to address health misinformation outside of the clinical setting include inoculation, debunking, and community-oriented motivational interviewing.^68,69^ Human rights organizations have advocated for transparent social media policies to facilitate access to reliable reproductive health information on social media.^18^

Beyond specific false claims, our findings reveal that misleading narratives about reproductive health pose a challenge to clinicians by undermining medical trust. Narratives may have greater persuasive power than claims and are highly context-dependent, but the studies we reviewed rarely distinguished between the two. While only 5% of statements directly undermined trust, many more did so indirectly by raising concerns about evidence-based medicine or recommending alternative interventions. Since most studies did not report on themes relating to trust, access to the underlying datasets is necessary to determine the extent to which narratives about trust are embedded in this content. Declining trust in medicine and public health in the USA has received considerable attention, especially in the context of the COVID-19 pandemic.^70^ Trust in reproductive healthcare and science is less frequently discussed, however. This discrepancy is notable given that trust in reproductive health is complicated by factors such as high maternal mortality rates in many countries including the USA, the historical exploitation of Black women in gynecology, and state reproductive coercion.^71^ The finding that trust-related concerns are represented on social media serves as a call-to-action for clinicians and institutions providing reproductive healthcare to build trust and exhibit trustworthiness. Reinforcing a patient-clinician relationship that has been weakened by online narratives requires acknowledging patients’ misinformed concerns and information sources.

A consistent theme across the misinformation examined in this study was the inflation of risks associated with medical interventions. In some cases, such as with contraception, side effects supported by the medical literature were disproportionately emphasized relative to benefits. For example, while the potential mental health harms of hormonal contraception were frequently discussed, little attention was given to the therapeutic effect of oral contraceptives on premenstrual dysphoric disorder. In contrast, for topics such as HPV vaccines, the risks that were mentioned had no scientific basis. This finding suggests that those who are averse to uncertainty or prone to fear may be more susceptible to reproductive health misinformation specifically in addition to other types of misinformation.^72^ Notably, fearful mindsets surrounding reproductive health are promoted by abstinence-only sexuality education, which often uses alarmist language to exaggerate risks associated with sexual activity.^73^ Comprehensive sexuality education, in contrast, has been proposed as an antidote to social media misinformation and information voids left by existing sex education.^74^

Non-evidence-based methods, often based in alternative and naturopathic perspectives, were suggested as alternatives to supposedly risky medical interventions. While alternative medicine has been extensively studied in oncology,^75^ less is known about its use in reproductive health. The growing prominence of the wellness industry in women’s health indicates the importance of better understanding its applications in reproductive health.^76^

Future research should contextualize these findings within patients’ overall information environments. It would be informative to learn how often patients encounter this misinformation, their responses to it, and to what extent they are exposed to reliable reproductive health information. This approach is important to understand the impact of crisis pregnancy center websites. While these sites were a prominent source of misinformation about contraception and abortion in this study, it is unclear who consumes this information and whether it influences reproductive health beliefs. Future studies can compare our findings to myths in non-digital spaces. Only three of the included studies analyzed artificial intelligence chatbots. These platforms are expected to increasingly provide health information, particularly for stigmatizing conditions.^77^ Future research should explore how patients interact with chatbots regarding reproductive health and mechanisms to improve the reliability of chatbot-generated reproductive health information. Evidence-based approaches to countering online misinformation, including community-oriented motivational interviewing,^69^ should be explored in reproductive health.

A narrative review approach allowed us to explore a comprehensive range of reproductive health claims and narratives. Without a systematic review methodology, however, articles that met our inclusion criteria may have been unintentionally excluded. While we assessed that thematic saturation was reached at the end of our search, it is possible that additional articles could contribute new insights. This study included papers published only in English. Since the studies we included typically focused on one topic in reproductive health and online platform, our findings do not necessarily reflect the most prominent reproductive health misinformation across social media overall, but rather the topics receiving the most research interest. For example, we did not find articles on menopause misinformation, although there is undoubtedly misinformation about menopause online. We are pursuing research to surveil online misinformation about reproductive health to understand its full scope.

In conclusion, we find that a variety of misleading claims and narratives about reproductive health are prominently reported in the literature. We urge clinicians providing reproductive healthcare to become knowledgeable about these misleading claims and narratives so that they can proactively assist patients in making decisions based on the best scientific evidence.

## Supplementary Information

Below is the link to the electronic supplementary material.

Supplementary file1 (DOCX 47.2 KB)
