# Supplementary material for: Online Misleading Information About Women’s Reproductive Health: A Narrative Review
Source: J Gen Intern Med. 2024 Nov 7;40(5):1123–31. doi: 10.1007/s11606-024-09118-6 (PMC11968640; doi:10.1007/s11606-024-09118-6)
Supplement: Supplementary file 1 — Supplementary file1 (DOCX 47.2 KB) [file 11606_2024_9118_MOESM1_ESM.docx]

**Appendix Table 1**

| **Topic** | **Sub-topic** | **Claim or narrative** | **Platform** | **Data source** | **Frequency** | **Misleading category** | **Citation** |
| --- | --- | --- | --- | --- | --- | --- | --- |
| Contraception and abortion | Contraception | The primary or only mechanism of IUDs is preventing implantation of a fertilized egg | Websites | Websites with information about IUDs | 24% | Inaccurate biological mechanism | Madden 2016^24^ |
| Contraception and abortion | Contraception | IUDs are an abortifacient | Websites | Websites with information about IUDs | 3% | Inaccurate biological mechanism | Madden 2016^24^ |
| Contraception and abortion | Contraception | Women in nonmonogamous relationships should not use IUDs | Websites | Websites with information about IUDs | 29% | Not aligned with professional guidelines | Madden 2016^24^ |
| Contraception and abortion | Contraception | Nulliparous women should not use IUDs | Websites | Websites with information about IUDs | 20% | Not aligned with professional guidelines | Madden 2016^24^ |
| Contraception and abortion | Contraception | Nonmonogamous nulliparous women should not use IUDs | Websites | Google search results for LARC | N/A | Not aligned with professional guidelines | Caddy 2023^25^ |
| Contraception and abortion | Contraception | Adolescents should not use IUDs | Websites | Google search results for LARC and adolescents | 16% | Not aligned with professional guidelines | Harris 2016^31^ |
| Contraception and abortion | Contraception | Hormonal contraception harms mental health | TikTok | Videos with hashtags #birthcontrolsideeffects and #nonhormonalcontraception | 24.3% | Exaggerated risk | Pfender 2024^30^ |
| Contraception and abortion | Contraception | Hormonal contraception harms mental health | YouTube | Vlogs from influencers about contraception | 32% | Exaggerated risk | Pfender 2023^22^ |
| Contraception and abortion | Contraception | Hormonal contraception causes weight gain | TikTok | Videos with hashtags #birthcontrolsideeffects and #nonhormonalcontraception | 16.9% | Exaggerated risk | Pfender 2024^30^ |
| Contraception and abortion | Contraception | Hormonal contraception causes weight gain | YouTube | Vlogs from influencers about contraception | 20% | Exaggerated risk | Pfender 2023^22^ |
| Contraception and abortion | Contraception | Combined oral contraceptives are associated with weight gain | Websites | Google search results for contraception | 18.80% | Unattributed risk | Marcinkow 2019^23^ |
| Contraception and abortion | Contraception | Combined oral contraceptives are associated with weight gain or loss | Websites | Google search results for contraception | 15.60% | Unattributed risk | Marcinkow 2019^23^ |
| Contraception and abortion | Contraception | Combined oral contraceptives delay fertility once discontinued | Websites | Google search results for contraception | 6.30% | Unattributed risk | Marcinkow 2019^23^ |
| Contraception and abortion | Contraception | Oral contraceptives increase risk of chlamydia and HIV | Websites | Websites of crisis pregnancy centers in Georgia | N/A | Unattributed risk | Swartzendruber 2018^28^ |
| Contraception and abortion | Contraception | Hormonal contraception causes changes in personality | TikTok | Videos with hashtags #birthcontrolsideeffects and #nonhormonalcontraception | N/A | Unattributed risk | Pfender 2024^30^ |
| Contraception and abortion | Contraception | Hormonal contraception causes changes in attraction | TikTok | Videos with hashtags #birthcontrolsideeffects and #nonhormonalcontraception | N/A | Exaggerated risk | Pfender 2024^30^ |
| Contraception and abortion | Contraception | Hormonal contraception causes digestive issues | TikTok | Videos with hashtags #birthcontrolsideeffects and #nonhormonalcontraception | 8.50% | Unattributed risk | Pfender 2024^30^ |
| Contraception and abortion | Contraception | Hormonal/chemical contraceptives increase the risk of chlamydia, HIV and STIs, and/or make the genital tract more vulnerable to infection | Websites | Websites of crisis pregnancy centers in Georgia | N/A | Unattributed risk | Swartzendruber 2018^28^ |
| Contraception and abortion | Contraception | Recommendations against using hormonal contraceptives | TikTok | Videos with hashtags #birthcontrolsideeffects and #nonhormonalcontraception | 5.60% | Discourages evidence-based interventions | Pfender 2024^30^ |
| Contraception and abortion | Contraception | Promotion of fertility awareness-based contraception | TikTok | Videos with hashtags #birthcontrolsideeffects and #nonhormonalcontraception | N/A | Promotes alternative medicine | Pfender 2024^30^ |
| Contraception and abortion | Contraception | Women discuss discontinuing contraception due to experience with or fear of side effects | TikTok | Videos with hashtags #birthcontrolsideeffects and #nonhormonalcontraception | 33.30% | Discourages evidence-based interventions | Pfender 2024^30^ |
| Contraception and abortion | Contraception | IUDs increase the risk of PID beyond the insertion month | Websites | Websites with information about IUDs | 27% | Unattributed risk | Madden 2016^24^ |
| Contraception and abortion | Contraception | IUDs decrease or cause infertility | Websites | Websites with information about IUDs | 14% | Unattributed risk | Madden 2016^24^ |
| Contraception and abortion | Contraception | Removing Mirena IUD after less than 5 years causes a "Mirena crash" | Websites | Google search results for LARC. | N/A | Exaggerated risk | Caddy 2023^25^ |
| Contraception and abortion | Contraception | Plan B and Ella cause ectopic pregnancy | Websites | Websites of Georgia crisis pregnancy centers | N/A | Unattributed risk | Swartzendruber 2018^28^ |
| Contraception and abortion | Contraception | Plan B has unknown risks relating to dependence, overdose, effects on pre-menarche and postmenopausal women, liver and kidney disease, and drug interactions | Websites | Websites of Georgia crisis pregnancy centers | N/A | Exaggerated risk | Swartzendruber 2018^28^ |
| Contraception and abortion | Contraception | Ella may be unsafe when taken by women who are younger than 18 or older than 35, use hormonal contraception, have already taken ella in the same cycle, or are pregnant or breastfeeding | Websites | Websites of Georgia crisis pregnancy centers | N/A | Exaggerated risk | Swartzendruber 2018^28^ |
| Contraception and abortion | Contraception | Hormonal contraception is unnatural | YouTube | Vlogs from influencers about contraception | 44% | Discourages evidence-based interventions | Pfender 2023^22^ |
| Contraception and abortion | Contraception | Contraception often causes side effects or adverse events | TikTok | Videos about contraception | N/A | Exaggerated risk | Stoddard 2024^27^ |
| Contraception and abortion | Contraception | Over-emphasizing side effects of the implant (61% mentioned unpredictable bleeding, 35% amenorrhoea, 30% significant pain during insertion, 30% negative impact on mood, 26% headaches, 22% weight changes) | YouTube | First-person testimonials about the contraceptive implant | 100% | Exaggerated risk | Paul 2017^29^ |
| Contraception and abortion | Contraception | Misleading promotion of cycle tracking as effective for contraception (not mentioning contraception that could be used during fertile windows or that the cycle tracking app Daisy retracted studies inflating efficacy) | YouTube | Vlogs from influencers about contraception | N/A | Promotes alternative medicine | Pfender 2023^22^ |
| Contraception and abortion | Contraception | Condoms are ineffective | Websites | Websites of North Carolina crisis pregnancy centers | 31% | Discourages evidence-based interventions | Bryant 2012^12^ |
| Contraception and abortion | Contraception | Condoms are ineffective, risky, or break often | Websites | Websites of crisis pregnancy centers with information about condoms or STIs that were listed in resource directories of states with mandatory waiting periods | 63.50% | Discourages evidence-based interventions | Bryant-Comstock 2016^13^ |
| Contraception and abortion | Contraception | Condoms are ineffective | Websites | Websites of Georgia crisis pregnancy centers | 78% | Discourages evidence-based interventions | Swartzendruber 2018^32^ |
| Contraception and abortion | Abortion | Abortion causes preterm birth | Websites | Websites of crisis pregnancy centers listed in state resource directories for pregnant women | 21% | Unattributed risk | Bryant 2014^33^ |
| Contraception and abortion | Abortion | Abortion causes preterm birth | Websites | Websites of North Carolina crisis pregnancy centers | 42% | Unattributed risk | Bryant 2012^12^ |
| Contraception and abortion | Abortion | Abortion causes breast cancer | Websites | Websites of crisis pregnancy centers listed in state resource directories for pregnant women | 20% | Unattributed risk | Bryant 2014^33^ |
| Contraception and abortion | Abortion | Abortion causes breast cancer | Websites | Websites of Georgia crisis pregnancy centers | 8% | Unattributed risk | Swartzendruber 2018^32^ |
| Contraception and abortion | Abortion | Abortion causes breast cancer | Websites | Websites of North Carolina crisis pregnancy centers | 11% | Unattributed risk | Bryant 2012^12^ |
| Contraception and abortion | Abortion | Abortion causes postabortion stress | Websites | Websites of crisis pregnancy centers listed in state resource directories for pregnant women | 73% | Unattributed risk | Bryant 2014^33^ |
| Contraception and abortion | Abortion | Abortion causes postabortion stress | Websites | Websites of North Carolina crisis pregnancy centers | 72% | Unattributed risk | Bryant 2012^12^ |
| Contraception and abortion | Abortion | Abortion causes mental health issues | Websites | Websites of North Carolina crisis pregnancy centers | 53% | Unattributed risk | Bryant 2012^12^ |
| Contraception and abortion | Abortion | Abortion causes mental health issues | Websites | Websites of Georgia crisis pregnancy centers | 36% | Unattributed risk | Swartzendruber 2018^32^ |
| Contraception and abortion | Abortion | Abortion causes mental health issues | Websites | Websites of crisis pregnancy centers listed in state resource directories for pregnant women | 48% | Unattributed risk | Bryant 2014^33^ |
| Contraception and abortion | Abortion | Medication abortion causes mental health problems | Websites | Google search results for medication abortion | N/A | Unattributed risk | Pleasants 2021^34^ |
| Contraception and abortion | Abortion | Abortion causes suicidal thoughts | Websites | Websites of crisis pregnancy centers listed in state resource directories for pregnant women | 26% | Unattributed risk | Bryant 2014^33^ |
| Contraception and abortion | Abortion | Abortion causes suicide | Websites | Websites of crisis pregnancy centers listed in state resource directories for pregnant women | 22% | Unattributed risk | Bryant 2014^33^ |
| Contraception and abortion | Abortion | Abortion causes infertility | Websites | Websites of crisis pregnancy centers listed in state resource directories for pregnant women | 13% | Unattributed risk | Bryant 2014^33^ |
| Contraception and abortion | Abortion | Abortion causes infertility | Websites | Websites of North Carolina crisis pregnancy centers | 19% | Unattributed risk | Bryant 2012^12^ |
| Contraception and abortion | Abortion | Medication abortion causes infertility | Websites | Websites of crisis pregnancy centers listed in state resource directories for pregnant women | N/A | Unattributed risk | Pleasants 2021^34^ |
| Contraception and abortion | Abortion | Abortion causes ectopic pregnancy | Websites | Websites of crisis pregnancy centers listed in state resource directories for pregnant women | 5% | Unattributed risk | Bryant 2014^33^ |
| Contraception and abortion | Abortion | Abortion causes miscarriage | Websites | Websites of crisis pregnancy centers listed in state resource directories for pregnant women | 6% | Unattributed risk | Bryant 2014^33^ |
| Contraception and abortion | Abortion | Abortion causes fetal pain | Websites | Websites of crisis pregnancy centers listed in state resource directories for pregnant women | 6% | Unattributed risk | Bryant 2014^33^ |
| Contraception and abortion | Abortion | Abortion causes placental previa | Websites | Websites of crisis pregnancy centers listed in state resource directories for pregnant women | 2% | Unattributed risk | Bryant 2014^33^ |
| Contraception and abortion | Abortion | Medication abortion leads to death | Websites | Websites of crisis pregnancy centers listed in state resource directories for pregnant women | N/A | Unattributed risk | Pleasants 2021^34^ |
| Contraception and abortion | Abortion | Medication abortion can be reversed | Websites | Websites of crisis pregnancy centers listed in state resource directories for pregnant women | N/A | Not aligned with professional guidelines | Pleasants 2021^34^ |
| Contraception and abortion | Abortion | Medication abortion can be reversed | Websites | Websites of Georgia crisis pregnancy centers | 3% | Not aligned with professional guidelines | Swartzendruber 2018^32^ |
| Contraception and abortion | Abortion | Medication abortion can be reversed | Facebook | Facebook Ad Library | Ads shown 18.4M times | Not aligned with professional guidelines | Center for Countering Digital Hate 2021^36^ |
| Contraception and abortion | Abortion | Medication abortion can be reversed | Facebook | Facebook Ad Library | Ads in 83% of searches for abortion | Not aligned with professional guidelines | Center for Countering Digital Hate 2021^36^ |
| Contraception and abortion | Abortion | Medication abortion can be reversed | Facebook | All public posts | 1,138 public posts | Not aligned with professional guidelines | Martiny 2022^17^ |
| Contraception and abortion | Abortion | Medication abortion can be reversed | Twitter | Tweets with the word "abortion" | N/A | Not aligned with professional guidelines | Sherman 2023^35^ |
| Contraception and abortion | Abortion | Ultrasound can predict miscarriage | Websites | Websites of Georgia crisis pregnancy centers that contain misinformation about miscarriage | 55% | Not aligned with professional guidelines | Swartzendruber 2018^32^ |
| Contraception and abortion | Abortion | Herbs can be used for abortion | Twitter | Tweets with the word "abortion" | N/A | Promotes alternative medicine | Sherman 2023^35^ |
| Contraception and abortion | Abortion | Herbs can be used for abortion | TikTok | Not specified | 102 videos with 18.1M views | Promotes alternative medicine | Sadeghi 2022^37^ |
| Contraception and abortion | Abortion | Herbs can be used for abortion | Facebook, Twitter, Instagram, websites | Fact-checked posts about women’s health across social media platforms | N/A | Promotes alternative medicine | Malki 2023^38^ |
| Contraception and abortion | Abortion | 15-25% of clinically recognized pregnancies end in miscarriage | Websites | Websites of Georgia crisis pregnancy centers | 17% | Other | Swartzendruber 2018^32^ |
| Contraception and abortion | Abortion | Abortion is never medically necessary (ending a pregnancy for a reason such as ectopic pregnancy is not an abortion) | Facebook | Not specified | N/A | Not aligned with professional guidelines | Macaluso 2022^11^ |
| Contraception and abortion | Abortion | Self-managed medication abortion is dangerous because a clinician is not involved | ChatGPT | Answers to questions about self-managed abortion | N/A | Exaggerated risk | McMahon 2024^39^ |
| Contraception and abortion | Abortion | Misoprostol should be administered orally in a medication abortion | ChatGPT and Google Bard AI | Answers to questions about abortion | N/A | Not aligned with professional guidelines | Mediboina 2024^40^ |
| Contraception and abortion | Abortion | The outcome of a medication abortion should be assessed with a home pregnancy test taken a few days to a week afterward | ChatGPT and Google Bard AI | Answers to questions about abortion | N/A | Not aligned with professional guidelines | Mediboina 2024^40^ |
| Contraception and abortion | Abortion | Tampons should be used to track bleeding following a medication abortion | ChatGPT and Google Bard AI | Answers to questions about abortion | N/A | Not aligned with professional guidelines | Mediboina 2024^40^ |
| Contraception and abortion | Abortion | Fever after a medication abortion is not a normal side effect | ChatGPT | Answers to questions about abortion | N/A | Other | Mediboina 2024^40^ |
| Contraception and abortion | Abortion | Patients experiencing pain during a medication abortion should take warm baths | ChatGPT and Google Bard AI | Answers to questions about abortion | N/A | Not aligned with professional guidelines | Mediboina 2024^40^ |
| Contraception and abortion | Abortion | Having multiple abortions increases the risk of an incomplete abortion | Google Bard AI | Answers to questions about abortion | N/A | Unattributed risk | Mediboina 2024^40^ |
| Contraception and abortion | Sex education | Marriage reduces the risk of STIs | Websites | Websites of crisis pregnancy centers with information about condoms or STIs that were listed in resource directories of states with mandatory waiting periods | 44.7% | Other | Bryant-Comstock 2016^13^ |
| Vaccines | Maternal vaccines | Maternal vaccines cause pregnancy complications (fetal loss, disability, or autism) | Twitter, forums, and blogs | Posts discussing maternal vaccines | N/A | Unattributed risk | Martin 2020^46^ |
| Vaccines | Maternal vaccines | The flu vaccine increases the risk of miscarriage | Twitter | Tweets about miscarriage and preterm birth | N/A | Unattributed risk | Cesare 2020^47^ |
| Vaccines | Maternal vaccines | The CDC and FDA do not provide accurate information about the safety of vaccines for pregnant women | Twitter, forums, and blogs | Posts discussing maternal vaccines | N/A | Undermines medical trust | Martin 2020^46^ |
| Vaccines | Maternal vaccines | Health institutions deceive women about maternal vaccines | Twitter, forums, and blogs | Posts discussing maternal vaccines | N/A | Undermines medical trust | Martin 2020^46^ |
| Vaccines | HPV vaccine | HPV vaccines can cause vaccine injury | Instagram | Posts with #HPV, #HPVVaccine, or #Gardasil hashtags | N/A | Unattributed risk | Massey 2020^41^ |
| Vaccines | HPV vaccine | HPV vaccines cause adverse health effects | Twitter | Tweets with #HPV hashtag | 59% | Unattributed risk | Kornides 2023^42^ |
| Vaccines | HPV vaccine | HPV vaccines cause death | Twitter | Tweets with #HPV hashtag | 23% | Unattributed risk | Kornides 2023^42^ |
| Vaccines | HPV vaccine | HPV vaccines have significant health risks | Websites | Search results for HPV vaccines from Google, Yahoo, Bing, and [Ask.com](http://ask.com/) | 12.40% | Unattributed risk | Madden 2012^44^ |
| Vaccines | HPV vaccine | HPV vaccines have psychological risks (e.g. increased sexual activity) | Websites | Search results for HPV vaccines from Google, Yahoo, Bing, and [Ask.com](http://ask.com/) | 6.70% | Unattributed risk | Madden 2012^44^ |
| Vaccines | HPV vaccine | HPV vaccines are ineffective | Twitter | Tweets with #HPV hashtag | 14% | Discourages evidence-based interventions | Kornides 2023^42^ |
| Vaccines | Covid vaccine | Pfizer Covid vaccine causes miscarriage | Facebook, Twitter, Instagram, websites | Fact-checked posts about women’s health across social media platforms | N/A | Unattributed risk | Malki 2023^38^ |
| Vaccines | Covid vaccine | Antibodies against SARS-CoV-2 spike protein developed in response to the mRNA vaccine can attach a placental protein (syncytin-1), causing infertility | N/A | Not specified | N/A | Unattributed risk | Abbasi 2022^45^ |
| Vaccines | Covid vaccine | The government and pharmaceutical industry cannot be trusted to provide safe Covid vaccines | Facebook, Twitter, Instagram, websites | Fact-checked posts about women’s health across social media platforms | N/A | Undermines medical trust | Malki 2023^38^ |
| Maternal health | Miscarriage | Progesterone therapy should be used to prevent miscarriage regardless of whether there is a history of recurrent pregnancy loss | Websites | Consumer websites for health information | N/A | Not aligned with professional guidelines | Ehrenreich 2019^49^ |
| Maternal health | Miscarriage | Lifestyle changes reduce miscarriage risk | Websites | Consumer websites for health information | N/A | Promotes alternative medicine | Ehrenreich 2019^49^ |
| Maternal health | Childbirth | Women should decline oxytocin administration | TikTok | Videos with #Pitocin hashtag | 35.40% | Discourages evidence-based interventions | Aaron 2023^51^ |
| Maternal health | Childbirth | Epidural analgesia carries risks including perineal tears, chronic pain, cesarean delivery, slurring of speech, oral herpes, and there is significant entry of epidural medications into the fetal bloodstream | YouTube | Videos about epidural analgesia | 92% | Unattributed risk | D'Souza 2021^52^ |
| Maternal health | Childbirth | The oxytocin infusion rate should be reduced | TikTok | Videos with #Pitocin hashtag | 28.10% | Discourages evidence-based interventions | Aaron 2023^51^ |
| Maternal health | Childbirth | Promoting mistrust in healthcare providers regarding oxytocin and/or fear of oxytocin | TikTok | Videos with #Pitocin hashtag | 37.50% | Discourages evidence-based interventions | Aaron 2023^51^ |
| Maternal health | Pregnancy | Women should not take psychotropic medications during pregnancy that their provider deemed necessary | [Babycenter.com](http://babycenter.com/) | Comments about the safety of the use of six common psychotropic medications during pregnancy | N/A | Not aligned with professional guidelines | Denton 2020^54^ |
| Maternal health | Pregnancy | Immediate cessation of nicotine during pregnancy is unsafe for the woman and baby | Discussion forums | Threads that discuss e-cigarette use during pregnancy | N/A | Unattributed risk | Wigginton 2017^48^ |
| Maternal health | Pregnancy | Women should gain more than the recommended amount of weight while pregnant | Websites | Google search results for weight gain during pregnancy | 18% | Not aligned with professional guidelines | Chang 2016^53^ |
| Maternal health | Pregnancy | Women should gain less than the recommended amount of weight while pregnant | Websites | Google search results for weight gain during pregnancy | 14% | Not aligned with professional guidelines | Chang 2016^53^ |
| Maternal health | Pregnancy | The herb ashwagandha should be used instead of Zoloft during pregnancy | [Babycenter.com](http://babycenter.com/) | Comments about the safety of the use of six common psychotropic medications during pregnancy | One post | Promotes alternative medicine | Denton 2020^54^ |
| Maternal health | Pregnancy | Women should take psychotropic medications that their provider warned them were unsafe during pregnancy | [Babycenter.com](http://babycenter.com/) | Comments about the safety of the use of six common psychotropic medications during pregnancy | N/A | Not aligned with professional guidelines | Denton 2020^54^ |
| Maternal health | Pregnancy | Women should use marijuana to cope with mental health problems during pregnancy | [Babycenter.com](http://babycenter.com/) | Comments about the safety of the use of six common psychotropic medications during pregnancy | N/A | Promotes alternative medicine | Denton 2020^54^ |
| Maternal health | Pregnancy | Women should strive for a "belly only pregnancy" where they do not gain excessive fat tissue | Instagram and blogs | Posts with #bellyonlypregnancy hashtag | N/A | Not aligned with professional guidelines | Steube 2022^50^ |
| Maternal health | Pregnancy | Doctors can't be trusted to give reliable information about psychotropic medications during pregnancy | [Babycenter.com](http://babycenter.com/) | Comments about the safety of the use of six common psychotropic medications during pregnancy | N/A | Undermines medical trust | Denton 2020^54^ |
| Maternal health | Pregnancy | Drug companies can't be trusted to provide information about psychotropic medications during pregnancy | [Babycenter.com](http://babycenter.com/) | Comments about the safety of the use of six common psychotropic medications during pregnancy | N/A | Undermines medical trust | Denton 2020^54^ |
| Fertility | IVF | Overrepresentation of live birth rate from IVF | TikTok | Videos with #IVF hashtag | Live births depicted in 89.3% of videos vs actual rate of 34.2% | Other | Peipert 2023^55^ |
| Fertility | Fertility preservation | Failure to mention that more than one cycle of egg retrieval may be needed to obtain enough mature eggs | Websites | Most used websites about oocyte cryopreservation | 86% | Not aligned with professional guidelines | Shao 2020^56^ |
| Fertility | Fertility preservation | Failing to mention the chance that women may not need to use their frozen eggs or the possibility that frozen eggs could fail to produce a viable pregnancy | Websites | Most used websites about oocyte cryopreservation | 86% | Not aligned with professional guidelines | Shao 2020^56^ |
| Fertility | General | X chromosome sperm last longer in the reproductive tract | TikTok | Videos with #IVF hashtag | N/A | Inaccurate biological mechanism | Peipert 2023^55^ |
| Fertility | General | AMH tests can estimate fertility and likelihood of conceiving | Websites | Websites selling direct-to-consumer AMH tests | 74% | Not aligned with professional guidelines | Johnson 2023^57^ |
| Fertility | General | AMH tests can predict menopause timing | Websites | Websites selling direct-to-consumer AMH tests | 74% | Not aligned with professional guidelines | Johnson 2023^57^ |
| Fertility | General | Low AMH levels can be treated, such as with vitamin D supplementation or stress reduction | Websites | Websites selling direct-to-consumer AMH tests | 7% | Promotes alternative medicine | Johnson 2023^57^ |
| Fertility | General | Women of all ages, not only those actively trying to conceive, should check their AMH levels | Websites | Websites selling direct-to-consumer AMH tests | 37% | Not aligned with professional guidelines | Johnson 2023^57^ |
| Breast cancer | Breast cancer | Mammography is harmful and "natural" alternatives are better | Twitter | Tweets discussing mammography | N/A | Promotes alternative medicine | Nastasi 2018^58^ |
| Breast cancer | Breast cancer | Foods can prevent or treat cancer | Pinterest | Posts mentioning breast cancer | 39.60% | Promotes alternative medicine | Wilner 2020^59^ |
| Breast cancer | Breast cancer | Supplements (e.g. iodine, slippery elm, colloidal silver) can prevent or treat cancer | Pinterest | Posts mentioning breast cancer | 19.80% | Promotes alternative medicine | Wilner 2020^59^ |
| Breast cancer | Breast cancer | Complementary and alternative medicine can treat breast cancer | Websites | Websites with information on breast cancer treatment | 11% | Promotes alternative medicine | Arif 2018^60^ |
| Chronic disease | PCOS | PCOS can be cured, especially with natural or herbal remedies (alkaline diet, orange tea, flaxseeds) | YouTube | Comments from videos about PCOS | N/A | Promotes alternative medicine | Malhotra 2023^61^ |
| Chronic disease | Endometriosis | Endometriosis can be treated exclusively with diet | N/A | Not specified | 39.2% of patients reported seeing this claim | Promotes alternative medicine | Arena 2022^4^ |
| Chronic disease | Endometriosis | People with endometriosis cannot become pregnant | N/A | Not specified | 54.6% of patients reported seeing this claim | Other | Arena 2022^4^ |
| Chronic disease | Endometriosis | People with endometriosis cannot become pregnant | TikTok | Videos about endometriosis | N/A | Other | Isaac 2024^62^ |
| Chronic disease | Endometriosis | Endometriosis can be caused by excess estrogen or ferritin | TikTok | Videos about endometriosis | N/A | Inaccurate biological mechanism | Isaac 2024^62^ |
| Chronic disease | Endometriosis | Androgenic hair growth and sensory issues can be signs of endometriosis | TikTok | Videos about endometriosis | N/A | Other | Isaac 2024^62^ |
| Chronic disease | Endometriosis | Endometriosis staging correlates with pain level | TikTok | Videos about endometriosis | N/A | Not aligned with professional guidelines | Isaac 2024^62^ |
| Chronic disease | Endometriosis | Endometriosis can be treated or cured with non clinically validated approaches, such as herbal remedies, acupuncture, juice cleanses, anti-inflammatory diets, or supplements. | TikTok | Videos about endometriosis | 14.10% | Promotes alternative medicine | Isaac 2024^62^ |
| Chronic disease | Endometriosis | Highlighting distrust in healthcare | TikTok | Videos about endometriosis | 24% | Undermines medical trust | Wu 2023^64^ |
| Chronic disease | Premature ovarian insufficiency | Patients with POI should consume foods with antioxidant and anti-inflammatory properties. | ChatGPT | Responses to questions about POI. | N/A | Promotes alternative medicine | Sütcüoğlu 2023^63^ |
| Chronic disease | Premature ovarian insufficiency | Genetic testing for POI is recommended for patients who have undergone chemotherapy or radiation therapy for cancer. | ChatGPT | Responses to questions about POI. | N/A | Not aligned with professional guidelines | Sütcüoğlu 2023^63^ |
| Chronic disease | Premature ovarian insufficiency | Antidepressant medications, such as selective serotonin reuptake inhibitors, can be used for sexual dysfunction symptoms associated with POI. | ChatGPT | Responses to questions about POI. | N/A | Not aligned with professional guidelines | Sütcüoğlu 2023^63^ |
| Chronic disease | Premature ovarian insufficiency | POI can be prevented by treating autoimmune diseases and through physical activity, diet, and stress management. | ChatGPT | Responses to questions about POI. | N/A | Promotes alternative medicine | Sütcüoğlu 2023^63^ |
| Other | Pelvic organ prolapse | Emphasizing surgical management of pelvic organ prolapse over more conservative options | YouTube | Videos about pelvic organ prolapse | N/A | Not aligned with professional guidelines | Herbert 2021^65^ |
